# Epigenetic strategies to reverse drug resistance in heterogeneous multiple myeloma

**DOI:** 10.1186/s13148-017-0319-5

**Published:** 2017-02-10

**Authors:** Mark E. Issa, Farnaz Sedigheh Takhsha, Chandra Sekhar Chirumamilla, Claudina Perez-Novo, Wim Vanden Berghe, Muriel Cuendet

**Affiliations:** 1 School of Pharmaceutical Sciences, University of Geneva, University of Lausanne, Rue Michel-Servet 1, CH-1211 Geneva 4, Switzerland; 2 Laboratory of Protein Science, Proteomics and Epigenetic Signaling (PPES), Department of Biomedical sciences, University of Antwerp, Campus Drie Eiken, Universiteitsplein 1, Wilrijk, Belgium

**Keywords:** Multiple myeloma, Epigenetic drugs, Stem cells, Drug resistance, Microenvironment, Treatment, Combination therapy

## Abstract

Multiple myeloma (MM) is a hematological malignancy, which remains incurable because most patients eventually relapse or become refractory to current treatments. Due to heterogeneity within the cancer cell microenvironment, cancer cell populations employ a dynamic survival strategy to chemotherapeutic treatments, which frequently results in a rapid acquisition of therapy resistance. Besides resistance-conferring genetic alterations within a tumor cell population selected during drug treatment, recent findings also reveal non-mutational mechanisms of drug resistance, involving a small population of “cancer stem cells” (CSCs) which are intrinsically more refractory to the effects of a variety of anticancer drugs. Other studies have implicated epigenetic mechanisms in reversible drug tolerance to protect the population from eradication by potentially lethal exposures, suggesting that acquired drug resistance does not necessarily require a stable heritable genetic alteration. Clonal evolution of MM cells and the bone marrow microenvironment changes contribute to drug resistance. MM-CSCs may not be a static population and survive as phenotypically and functionally different cell types via the transition between stem-like and non-stem-like states in local microenvironments, as observed in other types of cancers. Targeting MM-CSCs is clinically relevant, and different approaches have been suggested to target molecular, metabolic and epigenetic signatures, and the self-renewal signaling characteristic of MM CSC-like cells. Here, we summarize epigenetic strategies to reverse drug resistance in heterogeneous multiple myeloma.

## Background

Multiple myeloma (MM) is a form of hematological malignancy, which originates in the bone marrow (BM), accounting for 10% of hematological malignancy and 1% of the total cancer occurrence worldwide [1]. The annual incidence rate of MM in Europe alone is expected to be 4–6 cases/100,000 people/year mostly affecting older population with a median age of 63–70 years old [2]. MM is a heterogeneous disease that arises as a result of several disrupted cancer pathways, in particular those that promote clonal expansion of malignant plasma cells (PCs) and stimulate neoangiogenesis and osteoclastogenesis [3]. An excessive accumulation of myeloma PCs in the BM outperforms normal osteoblasts, which results into severe bone pain and contributes to the destruction of normal BM tissues [4, 5]. As a consequence, a set of defined symptoms appear in MM, which include excess of monoclonal PCs in the BM (>10%), monoclonal M proteins in the serum and/or urine, and myeloma-related impairments known as CRAB: C (calcium elevation), R (renal deficiency), A (anemia), and B (bone damage). Overexpression of surface antigens such as CD54, LFA-1, and CD56 by MM cells allows them to have complex and mutual interactions between the malignant PCs and the BM microenvironment. This promotes the secretion of paracrine cytokines, which lead to tumor cell survival, drug resistance and angiogenesis [6].

MM pathogenesis remains poorly understood, and the clinical response differs among MM patients due to the inter-individual variability and to the heterogeneous nature of the disease. Treatment options therefore vary based on the (epi)genetic profile of the MM patient, which are divided into high and standard risks. Modifications in the DNA sequence, compromising chromosomal transformations, deletions, and point mutations are thought to be crucial for the malignant transformation of PCs leading to MM [7]. However, the stratification of higher risk group from the lower risk group is ambiguous due to the lack of universally acclaimed prognostic markers representing the exact molecular heterogeneity of this disease.

### Epigenetic hallmarks of MM

**Fig. 1:**
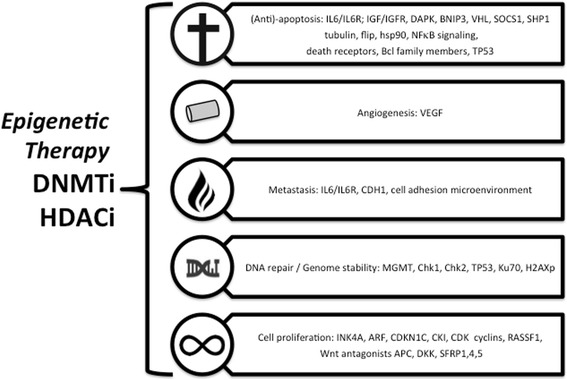
Epigenetic changes of specific target genes in MM related to cancer hallmarks (based on [8–16, 130, 131])

Recent studies indicated that in addition to genetic aberrations, epigenetic modifications directly contribute to MM development [8–13] (Fig. 1). Mechanisms of these modifications include the effects of microRNAs and those of polycomb proteins, DNA methylation, histone modifications, and chromatin remodeling [14–16]. In addition, genetic mutations of epigenetic modifier enzymes, and histone proteins identified by whole-exome sequencing approaches, further expand the epigenetic heterogeneity in MM [17] (Table 1). It is currently well known that the development of MM involves a slow progression of earlier events consisting of monoclonal gammopathy of undetermined significance (MGUS) followed by asymptomatic MM, which progressively evolves to symptomatic MM [18]. In contrast to other hematological malignancies such as chronic lymphoid leukemia (CLL), MM is not derived from one single driver mutation, suggesting that MM is more heterogeneous in disease manifestation [19, 20]. Furthermore, the spectrum of epigenetic modifier mutations in myeloma is broad with no single mutation present in a large proportion of patients [17]. All stages of MM (from MGUS to MM pathogenesis) share many features of a slow accumulation of cytogenetic abnormalities like mutations/deletions of chromosomal regions. Moreover, the frequency of mutations in epigenetic modifier genes encoding histone methyltransferases, histone acetyltransferases, and DNA (hydroxyl)methylation enzymes is significantly increasing upon cancer treatment [17]. Thus, from a basic biology standpoint, genetic alterations accompanied by epigenetic ones are the driving forces behind the MM pathogenesis [21]. Extensive research has shown that overall genome-wide hypomethylation pattern in cancer cells may lead to the reactivation of transposable elements and transcription modification of silenced genes [11, 22]. In contrast, DNA hypermethylation is responsible for the silencing of tumor suppressor genes in a variety of human malignancies, including MM [11]. The most important epigenetic change observed in MM is the global hypomethylation, which is associated with a poor disease prognosis [23]. Heuck et al. showed that myelomagenesis involved stage-specific alterations in DNA methylation suggesting that this phenomenon could be useful for distinguishing normal PCs from MGUS cells [9]. They also observed that while the early stages of MGUS were characterized by a hypomethylation status of tumor suppressor genes of B cells, when compared to normal PCs, the later stages were distinguished by a predominant hypermethylation reaching the maximum level in relapsed cases [9, 24, 25]. These findings indicate that an early demethylation in repetitive elements may be a potential destabilizing factor in MM pathogenesis, an effect that could promote secondary genetic events leading to the development of a full-blown disease [9]. Furthermore, gene-specific hypermethylation in 77 genes has also been identified during the transition of MGUS to MM [24, 25]. These genes are mostly tumor suppressor genes involved in developmental, cell cycle, and transcription regulation pathways [24, 25]. In MM patients, promoter hypermethylation of certain tumor suppressor genes including *p15*, *p16*, *VHL*, *XAF1*, *IRF8*, *TP53*, *CDKN2A*, *CDKN2B*, *DAPK*, *SOCS1*, *CDH1*, *PTGS2*, *CCND2*, and *DCC* was shown [26–33]. Of interest, DNA hypermethylation of the cell cycle inhibitors *p15* and *p16*, cyclin-dependent kinase inhibitor 2A (*CDKN2A*), and *TGFBR2* has been associated with poor prognosis in MM patients [27, 32, 34, 35]. Additionally, the most prominent DNA hypermethylation changes were observed in the 15% of patients with t(4;14) translocations, where the 4p16 break point occurred to the 5′ intron of multiple myeloma SET domain (MMSET), causing MMSET overexpression [36].

**Table 1:** Summary of genetic mutations in epigenetic writer-reader-eraser proteins or histones in MM

Mutations epigenetic writer, reader, or eraser enzymes	Epigenetic activity, mutation info Loss of function (LOF) Gain of function (GOF)	References
DNMT3A, TET2	DNA (hydroxy)methylation, LOF Reduced overall patient survival	[17, 40]
ARID1A/2/4A/5B	SWI-SNF chromatin remodelling, LOF	[17]
CHD2/4	Chromodomain, Missense, frameshift mutations, LOF	[17]
SETD2/B2	Histone H3K9 methylase, LOF	[17]
UTX/KDM6A	Histone H3K27 demethylase, Missense mutations, LOF Reduced overall patient survival	[17, 154]
UTY	Histone H3K27demethylase, LOF	[154]
EZH2	Histone H3K27 methylase, LOF	[155]
MMSET/NSD2/WHSC1	Histone H3K36K27 (de)methylase, translocation, point mutations, LOF/GOF	[156, 157]
MLL1/2/3/4/5	Histone H3K4 methylase, Missense/nonsense/frameshift/splice-site mutations, LOF	[53]
EHMT2	Histone H3K9 methylase, Missense mutations	[17]
KDM3B	Histone H3K9 demethylase, missense/nonsense/splice-site mutations	[17]
EP300/CREBBP	Histone acetylase, missense/nonsense/frameshift mutations, LOF	[17]
Histone mutations	Histone variant	Reference
HIST1H1B/C/D/E	Core/linker histone, Missense mutations, Frameshift insertion/deletion mutations	[17]

The major determinants of physiological DNA (hydroxy)methylation levels are the DNA methyltransferase (DNMT) enzymes [37], including DNMT1, DNMT3A and DNMT3B [38, 39], and DNA hydroxymethylase ten-eleven translocation (TET) enzymes. Whole-exome sequencing approaches in MM have recently identified various DNMT3A and TET2 mutations in MM [17, 40] (Table 1). However, the full extent of their involvement in the pathogenesis of MM disease and high risk behavior remains unclear [41]. Typically, transformation into MM is accompanied by progressive hypermethylation with maximum methylation seen in relapsed disease. Furthermore, it has been shown that the expression of DNMT1 within PCs from MM patients increased progressively and significantly through the disease course when compared to healthy PCs [42]. In contrast, DNMT3A and DNMT3B de novo methyltransferases were found to be underexpressed in both MGUS individuals and MM patients when compared to DNMT1 expression level [42, 43]. Although MM is characterized by widespread alterations in DNA methylation, DNA hydroxymethylation has also been observed in transcribed genes [43]. The cause of this aberrant expression of DNMTs is still unknown but it may be linked to the progressive increase in cell proliferation activity occurring in the various stages of the disease [44, 45]. It is already known that the expression level of DNMTs is “cell cycle” dependent and is elevated in cells with high proliferation rates [46, 47]. Altogether, DNA methylation changes in MM involve dynamic interplay of multiple signaling cascades, microRNAs (miRNAs) and non-coding RNA with DNMT and TET activities [48].

In contrast to DNA methylation, the landscape of histone modifications is more dynamic and constantly evolving [49]. Histones, and their modifications, are critical components of cellular programming and epigenetic inheritance. Structural changes in active euchromatin or silenced heterochromatin are controlled by chromatin writer, reader, and eraser enzyme complexes. They determine nucleosome positioning (histone octamers) along the DNA or reversibly modify (acetylation, phosphorylation, methylation, ubiquitination, glycosylation, sumoylation) histones on lysine, arginine, serine, or threonine residues of amino-terminal histone tails and establish specific chromatin states, which are involved in transcription regulation [50]. Recently, exome sequencing in MM has uncovered novel driver mutations in linker histones and multiple chromatin-modifying writer-reader-eraser enzymes, including H(D)MTs, HATs, ATP remodeling, and chromodomain proteins, spurring high interest how such mutations change enzyme activities or histone modification patterns and gene expression patterns in MM [16, 49, 51–53] (Table 1).

Histone deacetylases (HDACs) are dysregulated in MM with an aberrant overexpression of class I HDACs. This is correlated to a reduced overall survival of patients with MM [54]. MM cell lines also show a transcriptional upregulation of the histone methyltransferase (HMT) enhancer of zest homolog 2 (EZH2) as compared to healthy PCs which do not express EZH2 [55]. EZH2 belongs to a polycomb repressive group protein 2 (PRC2) complex that mediates silencing of gene transcription at the chromatin level through its HMT activity [56]. H3K27me3 is known to be methylated by EZH2 [56]. The increased EZH2 expression in MM cell lines may be mediated by interleukin 6 (IL6) in growth factor-dependent cell lines, caused by IL6-dependent c-Myc or Stat3 transcription factor activation controlling EZH2 transcription or via posttranscriptional miR-26a regulation [55, 57, 58]. Several studies have demonstrated the association between IL6 and proliferation response in MM cell lines [58–60]. In contrast to normal PCs that do not express EZH2, IL6 stimulation induced EZH2 protein expression in growth factor-dependent cell lines, while EZH2 was constitutively expressed in growth factor-independent cell lines [55]. The increased expression of EZH2 correlated with proliferation and B cell terminal differentiation [55].

Furthermore, the HMT MMSET protein is upregulated in all cases of MM with t(4;14) (p16;q32) translocations, accounting for approximately 15–20% of all patients with a poor prognosis [61, 62]. The HMT MMSET protein functionally interacts with corepressors and HDACs [63], catalyzing H4K20 trimethylation gene and loss of histone acetylation [63, 64]. Using the latest in Orbitrap-based technology, top-down mass spectrometry in MM patients with high and low MMSET expression has identified complex combinatorial H3 K14/K23 acetylation control of trivalent H3 K9/K27/K36 methylation marks [51, 52].

Additionally, MMSET enhances the function of HDAC 1 and 2, and the histone demethylase LSD1, suggesting that it is a component of corepressor complexes [63, 65]. Moreover, shRNA-mediated knockdown of MMSET was associated with the viability of MM cells [63]. This suggests a possible biological role of MMSET in malignant cell growth. Interestingly, MMSET plays a major role in constitutive activation of NF-κB, which is frequently deregulated in MM, by directly interacting with it and recruiting NF-κB target gene promotors, such as IL6, IL8, VEGFA, cyclin D, and Bcl-2 [66]. This leads to an elevation of histone H3K36me2 and H3K36me3 marks at the promoters resulting in their activation [66].

MiRNAs play a crucial role in the regulation of different cell functions, including cell differentiation, development, and apoptosis [67]. High throughput and functional studies have demonstrated aberrant miRNA expression in several human malignancies, where they can act as oncogenic molecules or as tumor suppressors, depending on their target transcripts [68–71]. MiRNAs also interact with important epigenetic regulators involved in the pathogenesis of MM. For example, inactivation of the tumor-suppressive miR-194-2192 cluster and miR-203 is associated with the pathogenesis of MM [72, 73]. These studies suggested that these miRNAs target the IGF pathway, preventing enhanced migration of PCs into BM. Furthermore, they are positive regulators for p53 and their downregulation plays an important role in MM development [74]. Pichiorri et al. compared the expression profile of miRNA in 49 MM cell lines, 16 BM CD138^+^s isolated from MM patients and 6 from MGUS patients, demonstrating that a common miRNA signature was associated with the multistep transformation process of MM [74]. Additionally, comparison of MGUS and MM samples with normal PCs, highlighted important miRNAs, including miR-32 and miR-17-92 cluster (located on chromosome 13), which were only upregulated in MM cells [75–77]. These miRNAs downregulated the expression of *SOCS-1*, which is frequently silenced in MM and plays an important role as inhibitor of IL6 signaling. MM patients with the deletion present on chromosome 13 showed a reduced survival rate [78]. Finally, miRNA expression may be regulated by DNA methylation and histone modifications [70]. Global miRNA suppression in MM could be due to hypermethylation of miRNA, such as miR-152, miR-10b-5p, and miR-34c-3p [79]. Re-expression of these miRNAs led to the suppression of oncogenes, inhibition of proliferation, and induction of apoptosis in MM cells, which suggested that miRNAs could act as potential tumor suppressors in this malignancy [79]. However, the mechanisms that control the expression of miRNAs are still mainly unknown.

### Epigenetic hallmarks of MM cancer stem cells

The most detailed characterizations of epigenetic alterations have been conducted in the whole cancer cell populations that form tumors or in cancer cell lines. The novel paradigm that tumors are composed of heterogeneous cell populations, namely tumoral cells and cancer stem cells (CSCs), imposes on the scientific community to address the specific epigenetic modifications in each cell population. In this part, the recent discoveries made regarding epigenetic modifications in CSCs, with a particular focus on MM, will be discussed.

The CSCs theory hypothesizes that a subset of tumor cells exhibits self-renewal properties and differentiation capabilities and is equipped with detoxification tools, such as ABC efflux transporters and aldehyde dehydrogenases [80], rendering those cells, named CSCs, highly resistant. CSCs are believed to be capable of replenishing the tumor and to be responsible for tumor relapse [81]. Using stem cell markers, including but not limited to CD24, CD34, CD44, CD133, and ALDH1, CSCs have been identified in virtually all cancers. However, although these CSCs markers have been demonstrated to identify cells capable of recapitulating the tumor in immunodeficient mice, heterogeneous cell populations have also been identified based on the use of these cell markers, suggesting that parental cells might undergo some changes during tumor progression [15]. For this reason, it has been suggested that CSCs should be identified based on functional assays. This stimulated a debate on the origin of CSCs and their role in tumor progression. Lineage tracing experiments conducted in adult murine normal stem cells (NSCs) demonstrated that NSCs exhibiting specific mutations represent the cancer cells of origin in skin, colon, leukemic, and brain tumors [15]. However, other reports indicated that CSCs can originate from more committed cells that acquire stem cell features, including self-renewal and differentiation capabilities [15]. This uncertainty in the origin of CSCs stimulated the search to understand how CSCs originate, their relationship to NSCs and other tumoral cells.

In order to understand the differences between CSCs and NSCs or between CSCs and tumoral cells, gene expression and transcriptional profiles were characterized and compared in each cell type. Developmental pathways, such as Hedgehog (HH), Notch, and Wnt/β-catenin, which control self-renewal and differentiation, were found to be extensively deregulated and subject to epigenetic alterations in CSCs. These pathways play pivotal roles in embryonic and tissue development. They are specifically involved in the regulations of NSCs, cell fate determination, and stem cell maintenance. Their deregulation contributes directly to tumor development, resistance, and metastasis. Epigenetic mechanisms involved in the regulation of CSCs, NSCs, and tumoral cells were investigated, and important observations have been made linking epigenetic alterations to survival advantages, tumor initiation, and resistance [15, 82].

HH signaling pathway is involved in the regulation of stem cell proliferation in various tissues, and alterations have been shown to contribute to tumor development [83, 84]. This pathway starts by the binding of a HH ligand to the patched-1 (PTCH-1) receptor, which results in the activation of smoothened (SMO), a transmembrane receptor. SMO, in turn, activates a family of transcription factors named GLI, which ultimately leads to the activation of target genes [84]. HH ligand promoted MM-CSCs (CD138^−^ cells) expansion with no effect on differentiation; whereas, HH pathway blockade with cyclopamine inhibited MM-CSCs expansion, diminished the clonal capacity of the MM cell lines NCI-H929 and KMS12, and decreased the CD138^−^ population through the induction of PC differentiation, suggesting that HH signaling plays a key role in the maintenance of MM-CSCs [83]. Various epigenetic variations of the HH signaling pathway have been described [85]. PTCH-1 promoter hypermethylation has been reported in several cancers, including gastric, ovarian, and breast cancers, causing a downregulation of the active form of the tumor suppressor PTCH-1 [85]. Treatment with 5-azacitidine (AZA) resulted in the unmethylation of PTCH-1 promoter, upregulation of PTCH1 expression, and apoptosis in gastric cancer cell lines [85]. Furthermore, the promoter HH-interacting protein (HHIP), another negative regulator of the HH signaling pathway, was found hypermethylated in liver cancer and pancreatic tumors, but no methylation was detected in adjacent healthy tissue (Table 2) [85]. In contrast, HH promoter is methylated in normal gastric tissue, but not in gastric carcinoma samples. Taken together, these results shed light on the importance of epigenetic deregulations in this pathway where tumor suppressor genes are deactivated and oncogenes activated during tumorigenesis.

**Table 2:** Summary of key epigenetic modifications found in MM and their therapeutic agent when available

Cancer cells	Epigenetic target/pathway	Epigenetic alteration	Therapeutic agent	Reference
Gastric	PTCH-1/HH	Hypermethylation	5-AZA	[85]
Liver, pancreatic	HHIP/HH	Hypermethylation		[85]
MM	JAG-2/Notch	Hyperacetylation		[88]
MB	HES1/Notch	miRNA-199b-5p	5-AZA	[89]
Colon	Notch1/Notch	miRNA-34a		[90]
MM	β-catenin/Wnt	Hypermethylation		[94]
MM	E-cadherin/Wnt	miRNA-23A		[16]
MM	HDACs	acetylation	PNB	[147]
MM	HDAC6	acetylation	RCL	[143]

*5-AZA* 5-azacytidine, *MB* medulloblastoma, *MM* multiple myeloma, *PNB* panobinostat, *RCL* ricolinostat

Notch signaling is an evolutionary conserved pathway crucial for normal development and growth. It consists of five ligands and four transmembrane receptors. The five ligands are Jagged (JAG)-1 and JAG-2, and Delta-like (DLL)-1, DLL-3, and DLL-4; and the four transmembrane receptors are Notch-1, Notch-2, Notch-3, and Notch-4. Upon binding of a ligand, a Notch transmembrane receptor is cleaved to produce the Notch intracellular domain (NICD). NICD then translocates to the nucleus where it acts to upregulate the expression of Notch target genes related to proliferation, differentiation, and survival [15, 86]. Aberrant activity of the Notch signaling pathway has been implicated in various neoplastic processes, including stem cell maintenance, metastasis, and angiogenesis. In MM, Notch activation promoted cell proliferation and accelerated disease progression; whereas, inhibition of Notch induced apoptosis, sensitized cells to chemotherapy, and prevented MM-induced osteoclast activation [87]. These studies highlight the importance of Notch signaling in maintaining MM-CSCs and in disease progression.

Epigenetic modifications affect the Notch signaling pathway at multiple levels, including ligands, receptors, and downstream effectors. In MM, the Notch ligand JAG-2 has been found overexpressed and the JAG-2 promoter region is aberrantly acetylated in MM cell lines and patient samples, an effect inflicted on histone acetylation and regulated by HDACs. Decreased SMRT levels were found in MM cell lines and patient samples, which resulted in an upregulation of Notch signaling. SMRTs are corepressors that typically recruit HDACs to promoter regions. The restoration of SMRT function induced JAG-2 downregulation as well as MM cell apoptosis. These results indicate a correlation between the acetylation status of the JAG-2 promoter and reduced levels of the SMRT corepressors in MM cell lines (Table 2) [88]. Notch pathway is also subject to regulation by miRNAs, which have been implicated in tumor growth, invasion, and metastasis. For instance, miRNA-199b-5p was found to negatively regulate HES1, a transcription factor involved in Notch signaling, an effect that negatively regulated the proliferation of medulloblastoma (MB) cells. In addition, overexpression of miRNA-199b-5p inhibited the expression of several stem cell specific genes, decreased the MB (CD133^+^) stem cell subpopulation, and inhibited the engraftment of MB cells in nude mice. In an analysis of 61 MB patients, miRNA-199b-5p expression was significantly higher in non-metastatic cases than that in metastatic ones and correlated positively with better overall survival. These data showing the downregulation of miRNA-199b-5p in metastatic MB suggest a potential silencing mechanism through epigenetic or genetic alterations. The use of 5-aza-2′-deoxycytidine, a DNA methylation inhibitor, resulted in a lower miRNA-199b-5p expression in a panel of MB cell lines, indicating an epigenetic mechanism of regulation of Notch in those cells (Table 2) [89]. In addition, microRNA-34a was shown as a tumor suppressor that regulated cell fate in early-stage dividing colon CSCs. Differentiating progeny exhibited higher levels of miRNA-34a; whereas, self-renewing CSCs displayed low levels. Interestingly, the balance between self-renewal and differentiating progeny was altered by miR-34a loss of function and gain of function experiments both in vitro and in vivo (Table 2). This phenomenon was found to be mediated by the sequestering actions of miR-34a on Notch1 mRNA, an effect that produced a threshold-like behavior where a Notch signal level determined self-renewal or differentiation. These findings demarcated an interesting feature in which miRNA regulated cell fate in CSCs [90].

The Wnt/β-catenin signaling pathway is involved in the regulation of self-renewal and differentiation of stem cells in several organs, including the bone marrow, skin, and intestinal crypt. This pathway includes 19 ligands and 10 receptors. The activation of the canonical pathway causes an accumulation of β-catenin in the cytoplasm, which in turn translocates to the nucleus and alters the expression of Wnt target genes. Aberrant Wnt signaling activity has been implicated in the development of several solid and hematological malignancies [91]. In MM, β-catenin was found to be constitutively activated, and the disruption of the active form of β-catenin appears as a promising therapeutic strategy in MM. The inhibition of Wnt/β-catenin downregulated the expression of Wnt target genes, induced cell death, inhibited tumor growth, and prolonged survival in mouse xenograft models [92]. This effect has been observed using either small molecules or siRNA directed towards β-catenin [91, 93]. Epigenetic dysregulation of the Wnt/β-catenin pathway has been demonstrated in MM. Gene silencing hypermethylation of multiple Wnt inhibitors (SFRP2, SFRP3, SFRP5, DKK3, APC, and WIF1) and of E-cadherin, an intracellular adhesion molecule responsible for cytoplasmic anchoring of β-catenin, were associated with constitutive activation of Wnt signaling in MM cells (Table 2) [94]. Furthermore, miRNA-23A, a miRNA that downregulated E-cadherin expression in lung cancer cells has been reported to be upregulated in MM. In addition, the promoter of miRNA-203, a direct inhibitor of the Wnt pathway that is frequently downregulated in MM, has been found hypermethylated in MM. In contrast, miRNA-21 and miRNA-200A were upregulated in MM; a situation that points out that the function of miRNA is context and cell type dependent (Table 2) [16].

### MM hallmarks of therapy sensitivity or drug resistance

Despite of the new therapeutic strategies for MM, including conventional chemotherapeutic agents, such as vincristine and doxorubicin, autologous stem cell transplant and novel agents such as bortezomib, thalidomide, and lenalidomide, MM remains predominantly incurable [95–97]. Depending on their stratification, patients are often subjected to treatments with frontline treatment options. The European medicines agency (EMA) considers oral combinations of glucocorticoids (prednisone), proteasomal inhibitors (thalidomide), and alkylating agents (melphalan) as a frontline therapy often followed by autologous transplantations [98]. Frontline therapies along with autologous transplantation have tremendously improved MM patients overall survival rate of 5 to 7 years, but MM patients often develop relapse over time and die of the disease in the refractory stage of treatment. There are several mechanisms that may play a role in MM resistance, including the bone marrow (BM) microenvironment, growth factors, genetic mutations, deregulated signaling pathways (e.g., multidrug resistance (MDR) genes, anti-apoptosis), clonal evolution of MM cells, and epigenetic gene inactivation [99].

Many MM relapsing patients who become resistant to treatment reveal increased expression of the MDR protein [100, 101]. MDR refers to a pleiotropic resistance to several structurally unrelated chemotherapeutic agents after exposure to a single cytotoxic drug [102]. Goldie and Coldman have hypothesized that the cause for this phenomenon is that a small number of potential resistant cells may be already present at diagnosis (before treatment) or may develop during treatment through spontaneous mutations, which later overgrow the sensitive cell population under the selective pressure of cytotoxic drugs [103]. MM has been shown to be generally MDR-negative at diagnosis. Almost 6% of newly diagnosed MM patients with no prior therapy had weakly expressed MDR markers. Relapsed MM patients are associated with a high expression of MDR 1 gene (*MDR1*, 43%) and P-glycoprotein (*P-gp*, 50–83%) [104]. P-gp is the first known member of ATP-binding cassette (ABC) transporter superfamily, which acts as an ATP-dependent efflux pump and is encoded by MDR1 gene [105]. The interaction of P-gp with its substrates results in the efflux of the substrates (e.g., doxorubicin, alkaloids) from the intracellular space to the extracellular space which causes decreasing of therapeutic efficacy [106]. Clinical trials with several P-gp inhibitors have demonstrated an increase of intracellular concentrations of affected drug, although the effects have been modest due to the lack of specificity and potency of those inhibitors [107].

Also, the BM microenvironment plays a fundamental role in drug resistance by regulating cell contacts between MM and BM stromal cells (BMSCs) and fibronectin adhesion, leading to cell survival, migration, and cell proliferation [108]. The mechanisms which lead to resistance due to the BM microenvironment in MM are soluble factor-mediated drug resistance (SFM-DR), including IL6 and cell-adhesion mediated drug resistance (CAM-DR), involving adhesion molecules (β1 integrins) [109]. Moreover, it has been described that MM cells express the receptor activator of NFκB (RANK) mRNA [110], and BMSCs and osteoblasts express the corresponding RANK ligand (RANKL) [111]. Binding of RANKL to RANK activates different signal transduction pathways in osteoclasts, including mitogen-activated protein kinase (MEK), extracellular signal-regulated kinase 1/2 (ERK1/2), phosphatidylinositol 3-kinase, (PI3K), Akt kinase, mammalian target of rapamycin (mTOR), and transforming growth factor β (TGF-β)-activated kinase [112]. These signaling pathways are crucial for proliferation, survival, apoptosis, and drug resistance in cancer cells [113]. Furthermore, CSCs identified in MM [114, 115], also referred to as cancer-initiating cells are resistant to chemotherapy, due to their ability to self-renew [116].

Furthermore, epigenetic alterations have been suggested to be involved in chemotherapy resistance in several cancer types including MM. Hypermethylation of tumor suppressor genes, including GPX3, RBP1, SPARC, and TGFB, may be involved in drug response and interaction with the BM [117]. Another study has shown that MM patients treated with bortezomib have a higher global DNA methylation, which is associated with a higher overall survival (OS) than patients with a low global DNA methylation [118]. The combination of highly methylated global genome with low NFKB1 methylation status defined a specific subset of patients with better prognosis [118]. Moreover, hypermethylation of CDKN2A, CDKN2B, TNF, and RB genes are more frequently shown in relapsed MM patients than in newly diagnosed patients [9]. Furthermore, Nojima et al. showed that methylation at the promotor region of *RASD1* gene in MM cells was correlated with its silencing and with reduced sensitivity to dexamethasone (DEX) [119]. Treatment of RASD1-hypermethylated MM cell lines with 5-aza-2′-deoxycytidine restored the expression of the gene and consequently the sensitivity to DEX [119]. In addition, alterations in chromatin modifications, such as histone methylation, are also involved in mediating chemotherapy resistance in MM. For example, anticancer drug-induced H3K27 hypermethylation is associated with CAM-DR in MM cells [120]. This is induced by H3K27 via inactivating phosphorylation of the transcription regulator EZH2 at serine 21, leading to the sustained expression of antiapoptotic genes, such as IGF1, B cell CLL/lymphoma 2 (BCL2), and hypoxia inducible factor 1, α subunit (HIF1A) [120].

Finally, the increased frequency of mutations detected in genes encoding for histone methyltransferases and DNA methylation modifiers in treated patients, suggests that these events may either play a role in disease progression or occur more frequently following exposure to induction chemotherapy in resistant subclones [17]. Hence, the use of sequencing-based diagnostics in myeloma at diagnosis, during cancer therapy and upon relapse may allow to identify potentially prognostic and/or targetable (epi)genetic lesions and provide potential new targets for personalized therapeutic strategies. Moreover, continuous efforts for counteracting the refractory stage of this disease and drugs with superior efficacy are urgently needed [121].

### Epigenetic strategies to overcome drug resistance in MM

Several studies have shown that beside genetic mutations, epigenetic alterations participated also in tumor growth and chemotherapy resistance [122, 123]. Epigenetic modifications are generally reversible, and this characteristic of allowing the malignant cell population to revert to a more “normal” state makes them an attractive therapeutic target. Chromatin-remodeling inhibitors targeting DNMTs, HMTs, HDACs, and bromodomain proteins or combinations thereof are currently being tested in various clinical trials for both cancer chemotherapies and cancer chemoprevention [124–126] (Table 3).

**Table 3:** Overview of published clinical trial studies of epigenetic drugs in MM

Epigenetic function	Compound	Phase	Reference
BET inhibitor	OTX015/MK-8628	I	[158]
BET inhibitor	CPI-0610	I	[159]
BET inhibitor	GSK525762	I/II	[159]
DNMT/HDAC inhibitor	Azacitidine/phenylbutyrate	III	[159]
HDAC inhibitor	Abexinostat	I	[159]
HDAC inhibitor	Belinostat	II	[159]
HDAC inhibitor	CI-994	II	[159]
HDAC inhibitor	CUDC-907	I	[159]
HDAC inhibitor	Entinostat	I	[159]
HDAC inhibitor	ITF2357	II	[159]
HDAC inhibitor	Panobinostat	II	[159]
HDAC inhibitor	Panobinostat in combination with bortezomib and dexamethasone	FDA approved	[146, 159]
HDAC inhibitor	Panobinostat in combination with everolimus	I/II	[159]
HDAC inhibitor	Rocilinostat	I/II	[159]
HDAC inhibitor	Tefinostat	I	[159]
HDAC inhibitor	Valproate	I	[159]
HDAC inhibitor	Vorinostat	I/II	[159]
HAT inhibitor	Curcumin	Preliminary clinical study	[160]

For example, DNMT inhibitors 5-aza-2'-deoxycytidine (decitabine; DAC) and 5-aza-2'-cytidine (AZA) appeared to be cancer cytostatic and cytotoxic as they trigger cell cycle arrest and DNA damage [127–129]. In MM cell lines, AZA showed anti-myeloma activity by p16 re-expression, caspase and PARP cleavage, and G0/G1-phase cell cycle arrest [130, 131]. Along the same line, DAC restored the expression of p15 by DNA methylation and induced a G0/G1- and G2/M-phase arrest linked with p21 and p38, respectively [132]. In addition, antiapoptotic pathways involving IL6 and NF-κB were suppressed by AZA [133]. Finally, latest investigations on the MM epigenome using genome-wide methylation arrays demonstrated the therapeutic benefit of DNMT inhibitors to reverse bortezomib or glucocorticoid drug resistance [101]. For example DNA hypermethylation in CDKN2A, CDKN2B, TNF, and RB genes has been detected more frequently in relapsed MM patients treated with bortezomib [101].

Recent studies showed that HDACs are promising targets for the treatment of MM, whereby significant in vitro cell death and in vivo tumor regression were detected [134]. The mechanisms by which HDAC inhibitors exert their effects in MM have been characterized and include the upregulation of cell cycle inhibitors, regulation of proapoptotic and antiapoptotic proteins, aggresome pathway activation, and proteasome inhibition. For instance, the HDAC inhibitor suberoylanilide hydroxamic acid (SAHA)-induced apoptosis in MM and B cell tumor cells, with increased p21 and p53 protein levels, dephosphorylation of Rb, and downregulation of Bcl-2. SAHA-induced cell death in a pattern indicative of calpain activation, and the calpain inhibitor calpeptin prevented SAHA-induced cell death, suggesting a mechanism by which HDAC inhibitors may exert their activity in MM [135]. Both SAHA and trichostatin A (TSA)-induced cell cycle arrest at the G1 phase and enhanced the apoptotic effects of TRAIL, a protein that induces apoptosis in MM cells. These effects of SAHA and TSA on the cell cycle were mediated by the upregulation of p21 and p27, and the inhibition of E2F transcriptional activity; whereas, the effects on apoptosis were mediated by the upregulation of Bim, Bak, Bax, Noxa, and PUMA, and downregulation of Bcl-2, Bcl-X, and IAPs. Interestingly, the apoptotic effects of TSA were increased by the proteasome inhibitor, MG132 [136]. Panobinostat (PNB), a hydroxamic acid derivative, in combination with bortezomib (BTZ), a proteasome inhibitor, resulted in a synergistic activity against DEX-sensitive and DEX-resistant MM cells, as well as in primary patient MM cells. BTZ and DEX are both used in combination as a first line therapy for MM (Tables 2 and 3) [137]. In the presence of BTZ, PNB induced α-tubulin hyperacetylation and caused aggresome formation. These results suggested a potential clinical benefit of combining proteasome inhibitors with HDAC inhibitors [138]. The precise mechanism of synergy is exerted by the dual inhibition of the proteasome and aggresome pathways, which results in increased levels of polyuquitinated proteins leading to cellular stress and apoptosis. The aggresome pathway is responsible for shuttling ubiquitinated proteins for lysosomal degradation. Specifically, proteasome inhibition leads to the accumulation of ubiquitin protein aggregates. The transport of protein aggregates along the microtubule network is facilitated by HDAC6, whose inhibition leads to synergistically increased cellular stress and apoptosis when used in combination with proteasome inhibitors [139]. Although this has been a central explanation for the synergistic effects, additional mechanisms have been characterized. For instance, preincubation with a subtoxic concentration of BTZ appeared to result in synergistic apoptosis induction in response to sodium butyrate and SAHA, two established HDAC inhibitors. The mechanism of this synergy was demonstrated as a BTZ-induced sensitization of U266 and MM.1S cells to sodium butyrate- and SAHA-induced mitochondrial dysfunction; caspase 9, 8, and 3 activation; and PARP degradation [140]. These effects were associated with NF-κB inactivation, p53 induction, caspase-dependent cleavage of p21, p27, Bcl-2, and X-linked inhibitor of apoptosis, and a marked ROS generation. Interestingly, the combination of bortezomib/HDAC inhibitors resulted in a pronounced CD138^+^ bone marrow cell death from MM patients, but this effect was not observed in the CD138^−^ cell population, suggesting a differential effect between tumoral PCs and MM-CSCs [140].

Although multiple HDAC inhibitors demonstrated important anticancer activities preclinically, their clinical utility has been limited due to adverse effects associated with pan-HDAC inhibition. Thus, isoform-selective inhibition may reduce those side effects. The inhibition of HDAC3 by knockdown or small-molecule inhibitor triggered significant MM cell growth inhibition via apoptosis. Importantly, HDAC3 inhibition, but not HDAC1 or 2, significantly augmented BTZ-induced cytotoxicity in vitro, and triggered tumor growth inhibition in a murine xenograft model of human MM, suggesting that HDAC3 represents a promising therapeutic target for the treatment of MM [141].

Furthermore, several HDAC6 inhibitors demonstrated important anticancer activities both in vitro and in vivo. HDAC6 is a zinc-dependent enzyme that belongs to class II histone deactylases [142]. An important HDAC6 target is α-tubulin, hence the important role of HDAC6 in protein trafficking, cell shape, and migration [142]. For these reasons, HDAC6 emerged as a valuable therapeutic target in cancer and other diseases [142]. HDAC6 inhibitors demonstrated strong antiproliferative activity, induced cell death in several cancer cell lines, and reduced tumor mass without overt toxicity [142]. These HDAC inhibitors include an important drug candidate termed ricolinostat (RCL). RCL, a hydroxamic acid HDAC6-selective inhibitor, showed strong anti-myeloma activity when combined with BTZ or with carfilzomib in preclinical studies. RCL selectively inhibited HDAC6, induced dose-dependent cell death in several sensitive and resistant MM cell lines, and triggered synergistic myeloma cell cytotoxicity when combined with BTZ or with carfilzomib both in vitro and in vivo (Table 2) [143, 144]. These promising results accelerated the examination of RCL in phase I/II clinical studies in R/R MM. Phase I clinical trials of RCL/DEX/lenalidomide in R/R MM demonstrated that RCL was safe and well-tolerated, and a preliminary examination showed that RCL exhibited significant antitumor activity in 55% of tested patients [145]. Phase I and II clinical trials, examining RCL in combination with pomalidomide and DEX, or in combination with BTZ and DEX, in R/R MM are ongoing.

PNB is a non-selective HDAC inhibitor that exerts a potent activity against all three classes of HDACs (I, II, and IV) (Table 2). PNB is an oral drug that was recently approved by the FDA for the treatment of patients with relapsed and/or refractory (R/R) MM who have previously been treated with at least two regimens (including BTZ, DEX, or immunomodulatory agents) [146]. Clinically, PNB is the first HDAC inhibitor to demonstrate a significant improvement in patients with R/R MM, and is currently prescribed in combination with BTZ and DEX. In R/R MM patients, PNB/BTZ/DEX was found to significantly prolong the progression free survival (PFS) in comparison to placebo/BTZ/DEX [146]. Importantly, PNB (when combined with BTZ/DEX) reduced the relative risk of death, relapse, and disease progression by 37% in R/R MM patients. In addition, PNB resulted in a more than 2-fold increase of the 2-year PFS, and the median follow-up by 1.15-fold [146]. Furthermore, the near-complete response and complete response was significantly higher in the PNB/BTZ/DEX group. Of note, the favorable results of PNB were consistently detected across different randomized control trials [147]. In general, the drug regimen PNB/BTZ/DEX exhibited a tolerable profile in R/R MM patients. The most frequent adverse effects were hematological, including thrombocytopenia (67%), lymphopenia (54%), and neutropenia (35%). The corresponding incidences of these adverse effects in the placebo group were 31, 40, and 11%. Common non-hematological adverse effects were also observed and included severe diarrhea (25%), pneumonia (13%), and peripheral neuropathy (18%). The corresponding incidences of these adverse effects in the placebo group were 8, 13, and 15% [146]. Clinical trials of PNB with carfilzomib or with lenalidomide/BTZ/DEX are ongoing for R/R MM, and desirable results are eagerly awaited. In conclusion, PNB (when combined with BTZ/DEX) appears a well-tolerated agent in patients with R/R MM and consequently a promising agent.

## Conclusions

Recently, outcomes for patients with MM have improved due to the application of “better developed” and novel epigenetic therapies. Although epigenetic drugs have significant anti-myeloma activity or can restore drug sensitivity [148], it remains unclear whether they can also restore the precancerous epigenetic state. In addition, inhibition of a specific epigenetic modifier may not kill the malignant CSC clone. Moreover, due to the complexity and epigenomic heterogeneity of MM cells, epigenomic profiling of the therapy resistant or sensitive MM cancer (stem) cell subpopulations may allow to personalize and optimize MM treatment protocols [149]. Finally, new combinations of frontline therapies with two or more epigenetic drugs may reveal additional synergistic or chemosensitising effects, pending an acceptable control of side effects [150–153].

## Abbreviations

AZA: 5-Azacitidine
BM: Bone marrow
BTZ: Bortezomib
CLL: Chronic lymphoid leukemia
CSCs: Cancer stem cells
DEX: Dexamethasone
DLL: Delta-like
DNMT: DNA methyltransferase
EMA: European medicines agency
EZH2: Enhancer of zest homolog 2
HDACs: Histone deacetylases
HH: Hedgehog
HHIP: HH-interacting protein
HMT: Histone methyltransferase
IL6: Interleukin 6
JAG: Jagged
MB: Medulloblastoma
MDR: Multidrug resistance
MGUS: Monoclonal gammopathy of undetermined significant
miRNAs: microRNAs
MM: Multiple myeloma
MMSET: Multiple myeloma SET domain
NICD: Notch intracellular domain
NSCs: Normal stem cells
PCs: Plasma cells
PFS: Progression free survival
PNB: Panobinostat
PRC2: Polycomb repressive group protein 2
PTCH-1: Patched-1
R/R: Relapsed and/or refractory
SAHA: Suberoylanilide hydroxamic
SMO: Acid; smoothened
TSA: Trichostatin A
